# Multiplexed digital spatial profiling of invasive breast tumors from Black and White women

**DOI:** 10.1002/1878-0261.13017

**Published:** 2021-06-10

**Authors:** Angela R. Omilian, Haiyang Sheng, Chi‐Chen Hong, Elisa V. Bandera, Thaer Khoury, Christine B. Ambrosone, Song Yao

**Affiliations:** ^1^ Department of Cancer Prevention and Control Roswell Park Comprehensive Cancer Center Buffalo NY USA; ^2^ Department of Biostatistics & Bioinformatics Roswell Park Comprehensive Cancer Center Buffalo NY USA; ^3^ Department of Biostatistics The State University of New York at Buffalo NY USA; ^4^ Department of Biostatistics and Epidemiology Rutgers School of Public Health Piscataway NJ USA; ^5^ Cancer Epidemiology and Health Outcomes Rutgers Cancer Institute of New Jersey New Brunswick NJ USA; ^6^ Department of Pathology Roswell Park Comprehensive Cancer Center Buffalo NY USA

**Keywords:** B7‐H3, breast cancer, digital spatial profiling, immune infiltrates, multiplex, racial disparities

## Abstract

The NanoString GeoMx digital spatial profiling is a new multiplexed platform that quantifies the abundance of tumor‐ and immune‐related proteins in a spatially resolved manner. We performed DSP for the simultaneous assessment of 52 analytes within spatially resolved tissue compartments defined by pan‐cytokeratin expression. We compared protein targets between 94 African American/Black and 65 European American/White cases, tumor and stromal tissue compartments, estrogen receptor alpha (ER)‐positive and ER‐negative cases, and explored potential biomarkers of survival. Of 33 analytes with robust signal for analysis, results were highly replicable. For a subset of markers, correlative analyses between DSP analytes and traditional immunohistochemistry scores revealed moderate to very strong associations between the two platforms. Similarly, DSP analytes and gene expression scores were concordant for 21 of 25 markers with overlap between the two datasets. Several analytes varied by ER status, and across the 25 immune markers surveyed, 14 had a significant inverse association with ER expression. B7 homolog 3 (B7‐H3; encoded by *CD276*) was the only analyte to show a significant difference by race, being lower in both the tumor and stromal compartments in Black women. DSP markers that were associated with survival included CD8, CD25, CD56, CD127, EpCAM, ER, Ki‐67, and STING. We conclude that DSP is an efficient tool for screening tumor‐ and immune‐related markers in a simultaneous fashion and yields results that are concordant with established immune profiling assays. DSP immune analytes were inversely associated with ER expression, in agreement with a substantial body of previous work that documents higher immune infiltration in ER‐negative breast cancers. This technology revealed that scores of the B7‐H3 protein were significantly lower in breast cancers from Black women compared with White women, an intriguing finding that requires replication in independent and racially diverse female populations.

## Graphical abstract

We evaluated the NanoString GeoMx digital spatial profiling platform for highly multiplexed immune profiling in a population of Black and White women with invasive breast cancer. Using DSP for discovery and conventional immunohistochemistry and gene expression data for validation, we found the DSP technology to be efficient, reproducible, and to yield results that are concordant with established immune profiling assays.

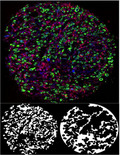

**Abbreviations**
DSP: digital spatial profiling
ER: estrogen receptor
FDR: Benjamini–Hochberg false discovery rate
FFPE: formalin‐fixed paraffin‐embedded
HR: hormone receptor
IHC: immunohistochemistry
PanCk: pan‐cytokeratin
ROI: region of interest
SNR: signal‐to‐noise ratio
TMA: tissue microarray
TME: tumor immune microenvironment
WCHS: Women's Circle of Health Study

## 1 Introduction

Immunotherapy has transformed patient care in some malignancies [1, 2, 3]. As a result, the characterization of the tumor immune microenvironment (TME) in a diverse array of cancer histologies has become an active area of oncology research. Recent technological advances in tissue staining have led to substantial progress in understanding the immune contexture in breast tumors [4, 5, 6]. One recent technology, the NanoString GeoMx digital spatial profiling (DSP) assay, combines standard immunostaining techniques with digital optical barcoding to achieve highly multiplexed immune profiling.

Digital spatial profiling can simultaneously quantify the abundance of more than thirty proteins in formalin‐fixed paraffin‐embedded (FFPE) tissues by counting unique oligonucleotide barcodes that are bound to antibodies and then released upon exposure to UV light. Barcodes are quantitated over a large dynamic range, and counts can be mapped to a region of interest (ROI), thereby allowing for digital profiles of analyte abundance in spatially distinct areas [7]. Due to its highly multiplexed nature and spatial resolution, DSP can be used to identify predictive biomarkers, reveal potential biological mechanisms of action, and characterize the abundance and distribution of key immune proteins in the TME in different populations of patients [8, 9, 10].

Previous tumor immune profiling studies were not typically conducted in racially diverse populations, and our understanding of the tumor immune contexture in breast cancer remains largely restricted to study populations of predominantly White women. Immune profiling studies can benefit from the inclusion of samples from Black women, as multimarker datasets for this group are still rare. The DSP technology is a unique opportunity to apply a sophisticated multimarker profiling approach to breast tumors from Black women and may ultimately improve immunotherapy prospects in this population.

We undertook the DSP technology to evaluate its effectiveness for screening invasive breast cancer tumors using samples from the Women's Circle of Health Study (WCHS), a multisite, case–control study designed to evaluate risk factors for aggressive breast cancer in African American/Black and European American/White women [11]. Here, our goal was to conduct a pilot study to quantify the abundance of tumor and immune‐related proteins in separate epithelial and stromal compartments of invasive breast cancer in women of Black or White ancestry, while also validating the DSP technology with two other methods we had previously applied to our study samples—conventional immunohistochemistry (IHC) and gene expression profiling.

## 2 Methods

### 2.1 Study population

The WCHS is a multisite, case–control study designed to evaluate the risk factors for aggressive breast cancer in Black and White women. Details on study recruitment have been described elsewhere [11, 12]. Briefly, participants were 20–75 years old; self‐identified as Black or White; had primary, histologically confirmed invasive breast cancer or ductal carcinoma in situ (DCIS); were diagnosed between 2001 to 2017; and had no previous history of cancer other than nonmelanoma skin cancer. Cases were first identified from several hospitals in New York City and then from 10 counties in New Jersey using rapid case ascertainment by the New Jersey State Cancer Registry. As part of the informed consent process, patients were asked to sign a release permitting the use of their tumor tissue blocks for research, and then tumor tissues and pathology reports were requested from treating hospitals. Clinicopathologic variables were extracted from the pathology reports: tumor size, grade, lymph node status, molecular subtype, and whether the patient received neoadjuvant therapy. Breast cancer subtypes were inferred from estrogen receptor (ER), progesterone receptor, and human epidermal growth factor receptor 2 (HER2) status on the pathology reports and were as follows: luminal A (HR^+^/HER2^−^), HER2‐positive (HR^+^ or HR^−^/HER2^+^), and triple‐negative (HR^−^/HER2^−^).

Tissue microarrays (TMAs) used for this study included FFPE tumor cores from invasive breast cancer that were selected by a board‐certified breast pathologist (TK) based on review of hematoxylin and eosin‐stained slides. TMA cores were 0.6 mm in diameter, and 75% of patient tumor samples were represented by at least two TMA cores (range 1–5 cores). Nine patients who received neoadjuvant therapy were excluded, leaving a final count of 159 cases (94 Black and 65 White). This study was approved by the Institutional Review Boards at Roswell Park Comprehensive Cancer Center and Rutgers Cancer Institute of New Jersey, and study methodologies conformed to the standards set by the Declaration of Helsinki.

### 2.2 Digital spatial profiling of breast tumor epithelial and stromal compartments

**Fig. 1:**
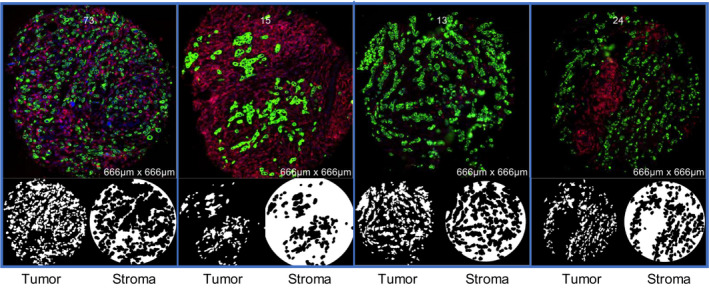
Schematic of multilabel immunofluorescence staining of four representative breast TMA cores. PanCk (green) served as a custom mask to differentiate tumor vs stromal ROIs.

The DSP assay (NanoString Technologies, Seattle, WA, USA) was performed as part of the NanoString Technology Access Program Service. Methods for DSP are described in detail elsewhere [7]. Briefly, FFPE TMA sections of invasive breast cancer cases were incubated with a cocktail of 58 barcoded antibodies, including three positive and three negative controls. A complete list of markers is provided in Table S1. Pan‐cytokeratin (PanCk) expression from an immunofluorescence assay was used as a custom mask to differentiate tumor and stromal compartments. Thus, each TMA core contained protein target scores for 2 ROIs—cytokeratin expressing regions of the core that were designated as tumor and regions that lacked cytokeratin expression that were designated as stroma (Fig. 1). Since most patients were represented by more than one TMA core, multiple tumor and stromal compartments per patient were averaged for each tissue compartment. Except for five cases that did not have a stromal compartment represented in the TMA core, each participant had two mean values for each of 58 analytes—one value for the tumor compartment and one for the stromal compartment.

UV light was applied to release the barcodes that were then collected, hybridized, and digitally counted using the NanoString nCounter instrumentation. Digital raw counts from barcodes corresponding to antibody targets were normalized to External RNA Control Consortium spike‐in controls to account for technical variation (e.g., hybridization efficiency). Normalized counts of each target were then evaluated relative to nonspecific counts from three negative isotype controls (Ms IgG1, Ms IgG2a, and Rb IgG), providing a signal‐to‐noise ratio (SNR) that accounts for signal variability due to ROI segment area and nonspecific binding of IgGs. We also assessed normalization based on housekeeping controls but found that the housekeeping proteins had low internal consistency and yielded results that were outside of our expectations for some well‐established markers. We applied a cutoff of 3 for the SNR to categorize markers that were likely to be below the lower detection limit. Nineteen experimental analytes with more than 50% of values having an SNR < 3 in either the tumor or the stromal compartment were deleted from our analysis. For the 36 remaining markers, SNR values < 3 were replaced with a single number 3/sqrt(2). Three of the 36 markers were positive controls, leaving 33 analytes of interest. Mean DSP values for each marker and participant are included in Table S2.

To evaluate the DSP assay reproducibility, we repeated assays on two separate days using adjacent TMA sections. We also evaluated DSP marker levels in fresh‐cut (Batch 1) vs stored (Batch 2) TMA sections. Because the SNR was higher for fresh‐cut TMA sections than stored sections (Fig. S1), we performed a calibration between the two batches using correction factors generated from a linear mixed‐effects model with batch as a random effect.

### 2.3 Immunohistochemistry and gene expression profiling

Women's Circle of Health Study TMAs were previously stained with ER, HER2, Ki‐67, CD4, and CD8 using conventional IHC; detailed methods pertaining to staining and quantitative scoring are described in earlier reports [12, 13, 14, 15]. Briefly, TMA slides were stained on an automated staining platform with monoclonal anti‐human antibodies against ER (clone SP1; Cell Marque, Rocklin, CA, USA), HER2 (clone CB11; BioGenex, Fremont, CA, USA), Ki‐67 (clone MIB1; Agilent, Santa Clara, CA, USA), CD4 (clone EP204; Biocare Medical, Pacheco, CA, USA), and CD8 (clone CD8/144B, Agilent). Slides were then digitally scanned using Aperio ScanScope (Leica Biosystems Inc., Buffalo Grove, IL, USA), and Aperio ImageScope quantitative image analysis software was employed to provide a digital score for each TMA core. Both IHC and DSP scores were log‐transformed, and the Pearson correlation coefficient (*R*) was used to assess the strength of the association between standard IHC and DSP scores. For HER2, which was scored on a categorical basis, ANOVA was used to test the hypothesis that the DSP scores for HER2 were the same for all IHC score categories (0, 1, 2, 3).

A subset of cases (*N* = 37) underwent gene expression profiling using the NanoString PanCancer Immune Panel in a previous study, and methodological details are described therein [15]. Briefly, whole section FFPE curls were used for RNA extraction and then 770 genes were sequenced using the nCounter assay performed by the Roswell Park Genomics Shared Resource and following the manufacturer's recommendations.

### 2.4 DSP marker levels and survival outcomes

We tested the associations between DSP scores and survival outcomes in 136 patients (85 Black, 51 White) with available follow‐up data. As described previously [12], data on vital status, including dates and causes of death, were available for cases enrolled in New Jersey through linkage with the New Jersey State Cancer Registry. The ICD‐10 code (C50) was used to identify breast cancer mortality. Follow‐up time was calculated from the date of enrollment to the last contact date (censored) or date of death. The median follow‐up time was 9.58 years [interquartile range (IQR) = 5.54 years], with 28 deaths, including 18 breast cancer‐specific deaths, as of October 31, 2018. Overall survival (OS), defined as the time from diagnosis to the date of the last contact or of death from any cause, and breast cancer‐specific survival, defined as the time from diagnosis to the date of the last contact or death from breast cancer, were analyzed with DSP scores dichotomized at the median, using Kaplan–Meier methods and log‐rank test.

### 2.5 Statistical analysis

Patient demographic and clinical characteristics were summarized overall and by race using the mean or median for continuous variables, and frequencies and relative frequencies for categorical variables. The expression levels of each marker were compared between Black and White cases, ER‐positive and ER‐negative cases, and tumor and stromal compartments. *T*‐Tests and nonparametric Wilcoxon rank‐sum tests were used as appropriate. The marker levels between the tumor and stromal compartments were compared using paired tests. For comparisons between Black and White patients, ANCOVA was conducted on the associations between race and marker values, adjusted for breast cancer subtype, grade, and BMI. The main effect of race was tested using sum of squares by F‐test. Model assumptions were checked using QQ and residual plots, and *P*‐values were corrected for multiple comparisons using the Benjamini–Hochberg false discovery rate (FDR) method. Correlation patterns among markers in the DSP panel were examined using unsupervised clustering, and heatmaps of analyte scores were constructed with the r package “ComplexHeatmap” version 2.4.2 [16]. Correlation plots were constructed with the r package “ggpubr” version 0.3.0. Other analyses were performed in sas version 9.4 (Cary, NC, USA) or r version 4.0.0.

## 3 Results

### 3.1 Descriptive characteristics of patients included in the DSP assays

Tumor tissues from 159 breast cancer patients, including 94 Black and 65 White women, were used for DSP assays. Patient demographic information and tumor clinicopathological characteristics are summarized in Table 1. Black patients had a significantly higher percentage of high‐grade, advanced stage, ER‐negative, and TNBC tumors than White patients, as well as a significantly higher prevalence of obesity.

**Table 1:** Patient demographic and primary invasive tumor characteristics among WCHS cases included in the DSP assay. PR, progesterone receptor. Four staging categories, I–IV, from the American Joint Committee on Cancer were examined, stage 0 patients were not included in this study. Low tumor grade denotes well‐differentiated tumors, intermediate denotes moderately differentiated, and high grade denotes poorly differentiated tumors. Tumor size (cm) was classified into three categories: < 1.0 cm, 1.0–2.0 cm, and > 2.0 cm. LN, lymph node status was defined as the presence (positive) or no (negative) cancer cells in axillary lymph nodes.

Variable	Categories	Overall	Black	White	*P*‐value
Overall		159 (100%)	94 (59.1%)	65 (40.9%)	
Age	Mean/(SD)	52.6/(10.2)	53.0/(10.7)	52.1/(9.6)	0.597
BMI	< 25	46 (29.3%)	19 (20.2%)	27 (42.9%)	**0.009**
	25–29	40 (25.5%)	27 (28.7%)	13 (20.6%)	
	30+	71 (45.2%)	48 (51.1%)	23 (36.5%)	
Subtype	Luminal A	97 (61.8%)	52 (56.5%)	45 (69.2%)	**0.005**
	HER2‐positive	26 (16.6%)	12 (13%)	14 (21.5%)	
	Triple‐negative	34 (21.7%)	28 (30.4%)	6 (9.2%)	
ER status	POS	112 (70.4%)	59 (62.8%)	53 (81.5%)	**0.018**
	NEG	47 (29.6%)	35 (37.2%)	12 (18.5%)	
PR status	POS	92 (57.9%)	46 (48.9%)	46 (70.8%)	**0.010**
	NEG	67 (42.1%)	48 (51.1%)	19 (29.2%)	
HER2 status	POS	26 (16.6%)	12 (13%)	14 (21.5%)	0.233
	NEG	131 (83.4%)	80 (87%)	51 (78.5%)	
Stage	I	65 (41.1%)	31 (33%)	34 (53.1%)	**0.028**
	II	72 (45.6%)	47 (50%)	25 (39.1%)	
	III/IV	21 (13.3%)	16 (17%)	5 (7.8%)	
Grade	LOW	22 (14.1%)	9 (9.8%)	13 (20.3%)	**0.001**
	MED	53 (34%)	24 (26.1%)	29 (45.3%)	
	HIGH	81 (51.9%)	59 (64.1%)	22 (34.4%)	
Tumor size	< 1.0	18 (11.5%)	8 (8.6%)	10 (15.6%)	0.056
	1–1.9	60 (38.2%)	31 (33.3%)	29 (45.3%)	
	≥ 2.0	79 (50.3%)	54 (58.1%)	25 (39.1%)	
LN status	POS	63 (41.2%)	42 (46.7%)	21 (33.3%)	0.138
	NEG	90 (58.8%)	48 (53.3%)	42 (66.7%)	

*P*‐values in bold are statistically significant.

### 3.2 DSP data QC metrices

Fifty‐eight protein markers, including three positive and three negative control markers, were assayed. Five TMA cores had no detectable stromal components, and thus only tumor ROIs were analyzed for these patients. The assays were performed in two batches, with the first batch of 2 TMA slides being freshly cut, and the second batch of 4 TMA slides was sectioned 3 years ago and stored in a desiccator. PCA analysis revealed apparent batch effects, which were subsequently corrected using data calibration (Fig. S2). Nineteen markers included in the panel had more than 50% of values below the lower detection limit (SNR <3) in either the tumor or the stromal compartment and were removed from our analysis, leaving 33 markers of interest with robust signal (Table S1). Based on DSP assays performed on two adjacent slides cut from each of the two TMAs in the first batch, strong correlations were observed, with a median rho of 0.970 (range 0.905–0.995) for the tumor compartment and 0.961 (0.858–0.992) for the stromal compartment.

### 3.3 Concordance between DSP and IHC

**Fig. 2:**
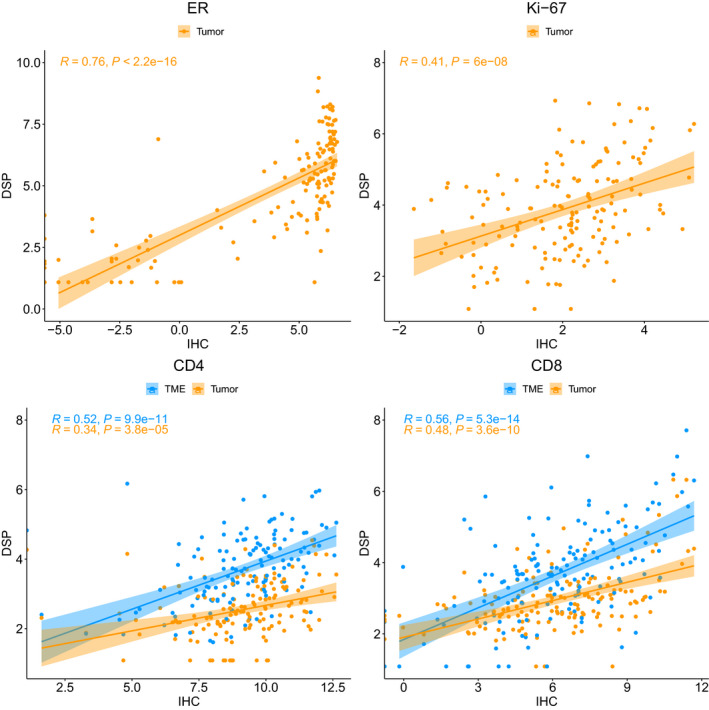
Correlation plots of log_2_‐transformed DSP and standard IHC scores for four common markers: ER, Ki‐67, CD4, and CD8. Tumor compartment scores were compared with IHC scores for ER and Ki‐67 because these markers express predominantly in the tumor. Scores for both the tumor and stromal compartments were evaluated for CD4 and CD8. The Pearson correlation coefficient (*R*) and corresponding *P*‐value for testing the hypothesis that true correlation is 0 were used to assess the strength of the association between standard IHC and DSP scores.

**Fig. 3:**
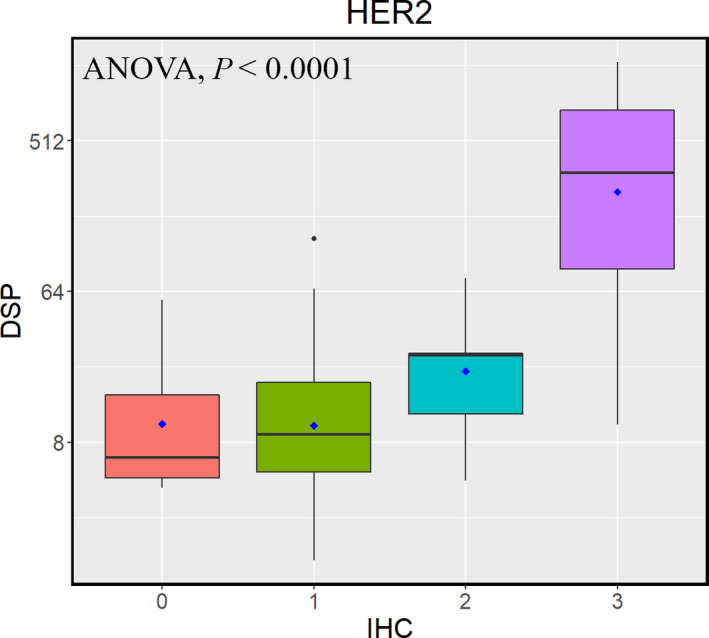
Boxplot of log_2_‐transformed DSP scores vs the categorical IHC scores for HER2 in the tumor compartment. ANOVA was used to test the hypothesis that the mean log_2_‐transformed DSP scores for HER2 were the same for all IHC score categories (0, 1, 2, 3) and the corresponding *P*‐value was reported.

Conventional IHC assays of ER, HER2, Ki‐67, CD4, and CD8 were performed on all patient samples in previously published work [12, 13, 14, 15]. Moderate to strong correlations were noted for these markers between the two platforms. DSP data from the tumor compartment were used for correlation analyses of ER, HER2, and Ki‐67, as these markers are predominantly expressed in the tumor, whereas DSP data from both the tumor and stromal compartments were evaluated for CD4 and CD8. As shown in Fig. 2, highly significant correlations (*P* < 0.0001 for all markers) were observed for ER (tumor *R* = 0.76), Ki‐67 (tumor *R* = 0.41), CD4 (tumor *R* = 0.34, stroma *R* = 0.52), and CD8 (tumor *R* = 0.48, stroma *R* = 0.56). Categorical IHC scores and DSP scores were also significantly associated for HER2 as shown with ANOVA (*P* < 0.0001; Fig. 3).

### 3.4 Concordance between DSP and gene expression data

**Fig. 4:**
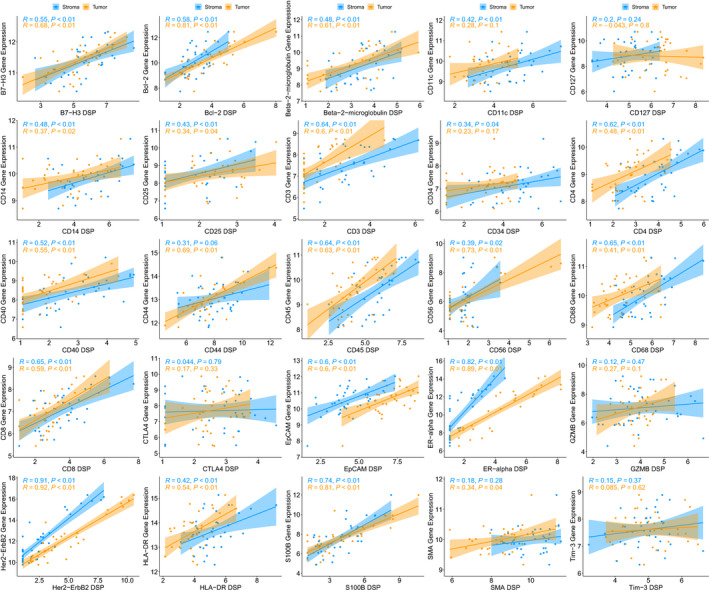
Correlation plots of log_2_‐transformed DSP scores and RNA‐seq scores for 25 markers that were present in both datasets. Gene expression data based on the NanoString PanCancer Immune Panel were available from a subset of the WCHS patients used for DSP assays (*N* = 37). The Pearson correlation coefficient (*R*) and corresponding *P*‐value for testing the hypothesis that true correlation is 0 were used to assess the strength of the association between gene expression and DSP scores. Of 25 markers, 21 markers showed moderate to very strong correlations between the two assays.

Gene expression data based on the NanoString PanCancer Immune Panel were available from a subset of the WCHS patients used for DSP assays (*N* = 37). Of 25 markers where data were available from both platforms, 21 markers showed moderate to very strong correlations between the two assays (Fig. 4). The strongest correlations were for Bcl‐2 (tumor, *R* = 0.81), CD56 (tumor, *R* = 0.73), ER (tumor, *R* = 0.89; stroma, *R* = 0.82), HER2 (tumor, *R* = 0.92; stroma, *R* = 0.91), and S100B (tumor, *R* = 0.81; stroma, *R* = 0.74), whereas moderate correlations were observed for B7‐H3 (tumor, *R* = 0.68; stroma, *R* = 0.55), Bcl‐2 (stroma, *R* = 0.58), β2‐microglobulin (tumor, *R* = 0.61; stroma, *R* = 0.48), CD11c (stroma, *R* = 0.42), CD14 (tumor, *R* = 0.37; stroma, *R* = 0.48), CD25 (tumor, *R* = 0.34; stroma, *R* = 0.43), CD3 (tumor, *R* = 0.60; stroma, *R* = 0.64), CD34 (stroma, *R* = 0.34), CD4 (tumor, *R* = 0.48; stroma, *R* = 0.62), CD40 (tumor, *R* = 0.55; stroma, *R* = 0.52), CD44 (tumor, *R* = 0.69), CD45 (tumor, *R* = 0.63; stroma, *R* = 0.64), CD56 (stroma, *R* = 0.39), CD68 (tumor, *R* = 0.41; stroma, *R* = 0.65), CD8 (tumor, *R* = 0.59; stroma, *R* = 0.65), EpCAM (tumor, *R* = 0.60; stroma, *R* = 0.60), HLA‐DR (tumor, *R* = 0.54; stroma, *R* = 0.42), and SMA (tumor, *R* = 0.34). No significant correlations were seen between DSP and gene expression assays for CD127, CTLA4, GZMB, and Tim‐3.

### 3.5 Differences in DSP marker levels between tumor and stromal compartments

**Fig. 5:**
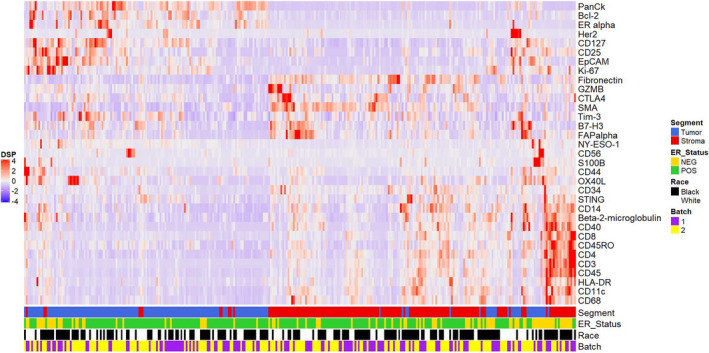
Heatmap depicting unsupervised clustering to summarize scores for immune analytes in tumor (PanCk positive) and stromal (PanCk negative) regions. Epithelial and stromal regions had analyte scores reflective of the cell types that are expected to be present in each region.

In general, the epithelial compartments had higher levels of tumor‐associated markers and the stromal compartments had higher levels of stromal and immune markers. Bcl‐2, CD25, CD127, EpCAM, ER, HER2, Ki‐67, NY‐ESO‐1, and PanCk were all significantly higher in the tumor compartment, whereas β2‐microglobulin, CD11c, CD14, CD3, CD4, CD8, CD34, CD40, CD45, CD45RO, CD56, CD68, CTLA‐4, FAPα, fibronectin, GZMB, HLA‐DR, OX40L, S100B, SMA, STING, and Tim‐3 were higher in the stromal compartment (Table 2). Unsupervised clustering illustrates that tumor and stromal compartments had distinct profiles (Fig. 5).

**Table 2:** Median analyte scores that were significantly different between the tumor and stromal compartments. Significance was determined using a paired *t*‐test with an FDR correction for multiple tests.

Marker	Tumor	Stroma	*q*‐value
	Median (IQR)	Median (IQR)	
Fibronectin	34.94 (44.52)	174.79 (123.96)	3.71E‐36
PanCk	439.06 (420.86)	22.33 (31.60)	6.97E‐36
SMA	392.49 (395.25)	1248.94 (1018.11)	3.13E‐28
CD127	69.42 (51.73)	30.22 (21.05)	3.93E‐27
EpCAM	94.09 (145.06)	17.75 (23.94)	4.63E‐24
CD4	5.66 (4.49)	13.29 (13.23)	3.18E‐21
CD45	14.12 (13.90)	39.29 (51.94)	1.01E‐18
CD14	14.29 (16.18)	26.38 (40.86)	1.26E‐18
CD11c	9.92 (6.64)	24.48 (22.30)	1.19E‐17
CD68	18.26 (12.49)	38.59 (35.30)	1.33E‐16
Ki‐67	14.39 (18.00)	5.62 (4.75)	7.06E‐16
Bcl‐2	22.28 (30.94)	8.91 (6.56)	2.81E‐15
CD3	2.12 (1.48)	6.52 (8.54)	1.26E‐14
FAPα	3.65 (3.83)	6.84 (6.61)	3.91E‐14
CD40	2.12 (1.79)	4.89 (5.37)	6.42E‐13
ERα	33.68 (74.16)	3.64 (6.20)	6.71E‐13
CD8	7.20 (3.75)	12.68 (13.34)	1.09E‐09
CD25	3.72 (3.52)	2.12 (2.53)	7.88E‐08
HLA‐DR	15.08 (13.76)	38.11 (40.62)	1.08E‐07
NY‐ESO‐1	8.26 (6.05)	3.94 (3.08)	6.41E‐06
CD45RO	4.86 (4.12)	5.8 (5.82)	2.11E‐05
HER2	11.20 (21.68)	3.32 (4.03)	5.37E‐05
CD34	10.01 (9.00)	22.89 (24.91)	1.01E‐04
GZMB	14.85 (9.89)	15.26 (13.67)	2.43E‐04
CTLA4	5.96 (4.29)	6.28 (6.83)	1.90E‐03
β2‐microglobulin	6.49 (7.42)	9.37 (8.72)	4.34E‐03
CD56	2.12 (1.60)	3.19 (1.55)	1.36E‐02
OX40L	3.83 (4.73)	3.93 (3.64)	1.53E‐02
S100B	7.31 (16.83)	10.90 (23.89)	1.53E‐02
Tim‐3	28.26 (22.69)	29.57 (18.74)	1.53E‐02
STING	11.55 (8.76)	15.47 (12.28)	2.39E‐02

### 3.6 Associations between ER and immune markers

**Fig. 6:**
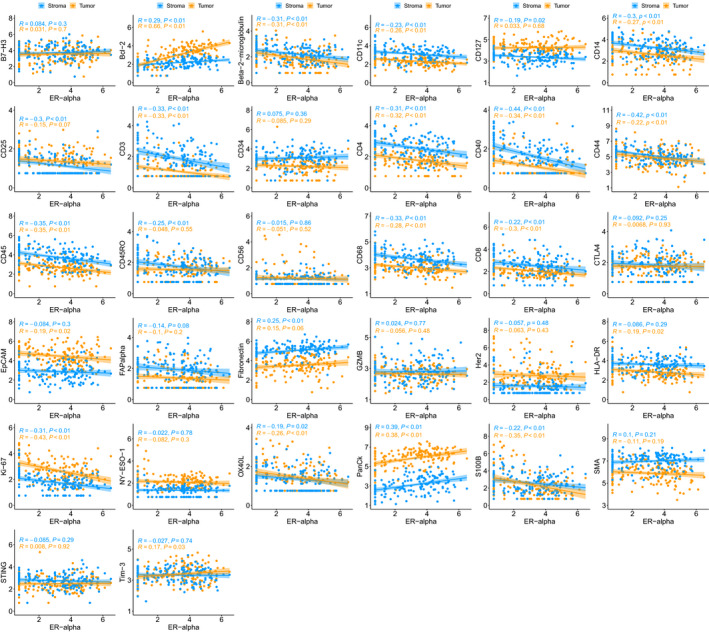
Correlation plots of log_2_‐transformed DSP data depicting the relationship between ER and other analytes in the panel. Most immune analytes are inversely associated with ER in both the tumor and stromal compartments. The Pearson correlation coefficient (*R*) and corresponding *P*‐value for testing the hypothesis that true correlation is 0 are shown.

Using ER status as assigned in the pathology report, we observed that several markers were differentially abundant between ER‐positive and ER‐negative cases. In general, these differences were in the expected direction, with significantly higher ER, Bcl‐2, Fibronectin, and PanCk scores in ER‐positive cases, and significantly higher β2‐microglobulin, CD3, CD4, CD8, CD11c, CD14, CD25, CD40, CD44, CD45, CD45RO, CD68, CD127, EpCAM, Ki‐67, OX40L, and S100B in ER‐negative cases (Table 3). When comparing markers between HER2‐positive and HER2‐negative cases, no significant differences were observed (Table S3). To further explore the potential interactions between ER and immune infiltration, ER measured in the tumor compartment by the DSP assay was analyzed as a continuous variable in relation to immune markers and revealed significant inverse associations, both in the tumor compartment: β2‐microglobulin (*R* = −0.31), CD3 (*R* = −0.33), CD4 (*R* = −0.32), CD8 (*R* = −0.30), CD11c (*R* = −0.26), CD14 (*R* = −0.27), CD40 (*R* = −0.34), CD45 (*R* = −0.35), CD68 (*R* = −0.28), and OX40L (*R* = −0.26); and in the stromal compartment: β2‐microglobulin (*R* = −0.31), CD3 (*R* = −0.33), CD4 (*R* = −0.31), CD14 (*R* = −0.30), CD25 (*R* = −0.30), CD40 (*R* = −0.44), CD44 (*R* = −0.42), CD45 (*R* = −0.35), and CD68 (*R* = −0.33; Fig. 6). These correlations were highly significant (*P* < 0.001). The only markers in the DSP panel to show a significant positive association with ER were Bcl‐2, Fibronectin, and PanCk.

**Table 3:** Median analyte scores for markers that had significant differential abundance between ER‐positive and ER‐negative cases. ER status was determined by the patient pathology report. *Q*‐values are presented for FDR‐corrected values that account for multiple tests.

Tumor				Stroma			
Marker	ER‐positive	ER‐negative	*q*‐value	Marker	ER‐positive	ER‐negative	*q*‐value
	Median (IQR)	Median (IQR)			Median (IQR)	Median (IQR)	
ER	49.46 (83.21)	3.19 (3.68)	3.7E‐11	ER	5.60 (7.00)	2.12 (0.00)	9.1E‐09
PanCk	514.71 (419.47)	225.88 (271.31)	4.1E‐07	PanCk	30.99 (37.46)	13.11 (12.64)	1.6E‐07
Bcl‐2	30.66 (29.87)	5.95 (8.41)	7.4E‐07	CD14	23.18 (26.72)	55.97 (56.26)	1.2E‐04
Ki‐67	10.53 (11.69)	26.56 (38.69)	1.9E‐05	CD40	3.93 (4.54)	8.89 (10.34)	1.2E‐04
CD45	12.26 (9.62)	21.11 (31.75)	3.4E‐03	β2‐microglobulin	8.14 (7.12)	13.92 (11.75)	9.6E‐04
CD11c	9.34 (5.50)	13.08 (14.8)	3.5E‐03	CD25	2.12 (1.80)	4.63 (3.02)	9.6E‐04
CD14	12.37 (12.03)	24.05 (32.89)	3.5E‐03	CD45	34.34 (39.87)	76.24 (94.88)	9.6E‐04
CD40	2.12 (1.05)	3.92 (5.42)	3.5E‐03	CD127	27.47 (18.86)	38.12 (21.47)	2.9E‐03
CD68	16.67 (10.37)	23.34 (21.67)	7.4E‐03	CD4	10.90 (10.93)	18.19 (19.56)	3.9E‐03
β2‐microglobulin	5.63 (5.41)	10.96 (12.22)	7.6E‐03	CD68	33.53 (25.39)	56.9 (45.60)	3.9E‐03
CD4	5.19 (3.51)	8.12 (6.62)	1.0E‐02	Ki‐67	5.02 (3.49)	8.64 (6.38)	4.8E‐03
EpCAM	68.16 (99.55)	160.71 (134.80)	1.0E‐02	CD45RO	5.03 (4.12)	8.14 (9.40)	5.1E‐03
S100B	5.33 (10.86)	32.85 (128.89)	1.0E‐02	S100B	8.77 (19.23)	28.22 (43.11)	7.1E‐03
OX40L	3.69 (3.01)	4.85 (6.55)	1.2E‐02	CD3	5.65 (6.31)	8.4 (17.43)	8.5E‐03
CD25	3.52 (2.57)	4.91 (4.00)	1.2E‐02	CD11c	20.62 (20.26)	30.52 (23.77)	1.0E‐02
Fibronectin	38.54 (50.70)	28.03 (28.73)	2.0E‐02	CD44	130.01 (147.33)	268.76 (309.84)	1.5E‐02
GZMB	13.88 (9.26)	17.08 (11.11)	2.7E‐02	Fibronectin	186.98 (119.64)	137.65 (109.95)	2.2E‐02
CD8	6.6 (3.69)	8.44 (9.27)	2.9E‐02	OX40L	3.58 (3.17)	4.38 (4.03)	2.2E‐02
CD3	2.12 (0.98)	3.08 (3.88)	3.8E‐02				

### 3.7 Differences in DSP markers between Black and White breast cancer patients

After adjustment for tumor subtype, grade, and BMI, B7‐H3 was the only marker to show a significant difference by race (Tables S4 and S5), with Black women having lower scores in both the tumor and stromal compartments (tumor: median 29.35 vs 51.01, *P* = 0.0001; stroma: median 34.62 vs 61.71, *P* = 0.0002). The differences remained significant after FDR correction for multiple comparisons (tumor: *q* = 0.0018; stroma *q* = 0.0057). Within Black breast cancer patients, B7‐H3 levels were significantly lower in less aggressive tumors, including stage I (*P* = 0.0298), low grade (*P* = 0.0421), and tumors < 1 cm in size (*P* = 0.0003). A similar trend was observed in White patients. Gene expression data from the NanoString PanCancer Immune Panel also showed lower levels of B7‐H3 in Black than White patients (log_2_‐fold change = −0.136, unadjusted *P* = 0.0122, FDR adjusted *q* = 0.0673).

### 3.8 DSP analytes and survival outcomes

In exploratory univariate analysis, we found three markers associated with OS in the tumor compartment (EpCAM, *P* = 0.0013; Ki‐67, *P* = 0.034; STING, *P* = 0.036) and three in the stromal compartment (CD8, *P* = 0.028; CD25, *P* = 0.013; CD127, *P* = 0.0059; Figs S3 and S4). For breast cancer‐specific survival, four markers were associated with OS in the tumor compartment (EpCAM, *P* = 0.0072; ER, *P* = 0.037; Ki‐67, *P* = 0.039; STING, *P* = 0.043) and four in the stromal compartment (CD8, *P* = 0.024; CD25, *P* = 0.013; CD56, *P* = 0.018; ER, *P* = 0.018; Figs S5 and S6). No significant associations were observed between B7‐H3 and OS or breast cancer‐specific survival in the overall population, or when Black patients were analyzed as a separate group.

## 4 Discussion

We used NanoString GeoMx DSP as a new approach to characterize the breast tumor microenvironment in a subpopulation from the WCHS and to investigate immune differences due to race and tumor histopathological characteristics. For our sample set, DSP quantitated protein targets in separate tumor and stromal compartments and allowed the simultaneous assessment of 33 markers with robust SNRs. The DSP system quantified direct digital counts of antibody abundance over a large dynamic range and reproducibility was high when adjacent TMA sections were compared. Moreover, we found moderate to high concordance with IHC and gene expression assays for several markers. DSP was a straightforward and efficient tool in this discovery study, revealing interesting findings that may warrant further investigation.

Digital spatial profiling overcomes the major limitations of traditional IHC and immunofluorescence approaches, principally the capability to interrogate many markers simultaneously on a single slide. In the current study, we were able to simultaneously evaluate 33 markers with high confidence. Quantitative IHC scores that were previously reported for five markers in our dataset—CD4, CD8, ER, HER2, and Ki‐67—provided an opportunity to evaluate the concordance between DSP and standard IHC staining in our samples, albeit on different sections from the same TMA block. We found moderate to strong agreement with correlation coefficients in the same range as previous studies [10, 17, 18]. Imperfect agreement may be explained by antibody performance differences between the multiplex vs single‐plex format, antibody clones, staining conditions, preanalytical variables, and section to section variability between TMA slices. We further investigated the concordance between DSP and gene expression, using the NanoString PanCancer Immune Panel. Similar to our comparisons with IHC, we observed that analyte measurements obtained with DSP were largely congruent with those based on RNA expression.

Our previous work has shown that ER status is a strong stratifying factor for immune expression in breast tumors, with ER‐positive cancer being immunologically “cold” and ER‐negative cancer being immunologically “hot” [12]. ER expression is one of the most important clinicopathologic features of breast cancer, but the interaction between ER and immune cells in the breast TME is not fully understood. It is established that ER‐negative breast tumors have higher levels of immune infiltration than ER‐positive tumors [19, 20, 21, 22, 23], but less is known about the mechanisms that cause breast tumors to be highly infiltrated with immune cells vs immune‐excluded. Estrogens and ERs can modulate both innate and adaptive immune functions, and previous work suggests a potential immune regulatory role of estrogens in the breast tumor microenvironment [24, 25, 26, 27]. DSP revealed that ER levels in the tumor compartment were inversely correlated with a wide array of immune markers, suggesting that ER expression may act to somehow diminish immune cells or their activity in the breast TME. Unlike most previous studies that relied on a dichotomized ER status (positive vs negative), the multiplexed DSP panel allowed us to perform a more in‐depth analysis using a continuous scale to show the dynamics of tumor ER expression and immune infiltration in separate stromal and tumor compartments.

Digital spatial profiling was useful for screening tissue compartments for various immune analytes and identified B7‐H3 as being significantly less abundant in Black women, in both tissue compartments. B7‐H3 (CD276) is a member of the B7 superfamily with roles in innate and adaptive immunity and also has nonimmunologic functions [28, 29, 30]. Early work reported a costimulatory role for B7‐H3 [28], but subsequent reports have shown negative immunoregulatory and inhibitory functions [31, 32, 33]. To date, the precise physiological roles of B7‐H3 are not fully elucidated and its receptor has yet to be identified [30]. B7‐H3 is aberrantly expressed in a number of cancers, including breast cancer, and is generally associated with poor prognosis and clinical outcome [29, 30, 34]. In a separate study of a racially diverse population, Black patients with colorectal cancer had significantly lower expression levels of B7‐H3 than Whites, and B7‐H3 was a negative prognostic biomarker [35].

B7‐H3 may be a promising target for therapeutic interventions because it is aberrantly expressed in large number of solid tumors and tumor vasculature, but has limited expression in normal tissues [30, 36]. Recent work shows CAR‐Ts that target B7‐H3 in solid tumors can control tumor growth, both *in vitro* and in mouse models [37]. A separate report shows anti‐B7‐H3 drug conjugates display potent tumoricidal effects, killing both cancer cells and tumor vasculature [36]. Importantly, B7‐H3 occurs in both the tumor and stromal compartments in triple‐negative breast cancer, and thus may be a target for this subtype that has limited therapeutic options [36]. To improve the success of immunotherapeutic agents in breast cancer, future work of the immune response in breast tumors and the interplay with surrounding microenvironmental features will be crucial for understanding mechanisms of immune evasion and tumor resistance in different patient populations. Sophisticated immune profiling technologies can aid these research endeavors, as demonstrated in our study using the DSP platform.

Differences in host immune responses are well documented in populations of African and European ancestry [38, 39, 40, 41], and there is a growing body of literature that indicates the tumor immune environment in breast tumors may be an area of divergence between Black and White women [12, 42, 43, 44, 45, 46]. This line of work, typically based on either single‐stain or double‐stain IHC assays, RNA expression, or DNA sequence analyses, has shown that Black women with breast cancer have more prominent interferon signatures, lymphocytes, macrophages, MHC1 metagene expression, higher immune dysfunction scores, and lower expression levels of PD‐L1 and mast cells [12, 42, 43, 44, 45, 46]. We therefore expected that within the TME, divergence in immune profiles between Black and White women would occur. However, we note that DSP did not corroborate previously published reports that used immunostaining methods to show that CD8^+^ T cells and macrophages were significantly more abundant in Black women [12, 42, 43, 45]. There are several possibilities why this occurred, such as the relatively small sample size of the current study, differences in antibody clones, or the inability to fine‐tune antibody binding conditions when so many markers are batched together in a one‐size‐fits‐all staining procedure [17]. Steric hindrance has been reported when antigens have biological overlap, such as when CD8 is combined with CD3, as in our panel [47]. Steric hindrance and multistain optimization constraints accompany any large multiplexed assay and likely deserve more consideration in data interpretation than is currently allotted. Future DSP studies with larger sample size may corroborate previous findings pertaining to CD8^+^ T cells and macrophages, or it may be necessary to reexamine assay conditions for some markers in the current DSP panel.

Other limitations of DSP include those that can occur in any study of archived FFPE samples, that is, preanalytical variation in diverse samples and nonspecific antibody binding. Spatial resolution, while informative in the context of our study, was limited to the overall tissue compartment, and fine spatial details such as the distances between different markers are not readily accomplished with the DSP platform. Due to low SNRs, 19 of 58 markers in the panel had to be deleted from the analysis. This could be due to low expression of these targets in breast tumors or explained by suboptimal antibody performance in large multiplexing assays as described above. Without further assays, the cause of the low SNRs is unknown and our interpretation and conclusions drawn from these markers are curtailed. Zugazagoita *et al*. [48] also reported low SNRs in a subset of markers in a separate DSP study with lung cancer samples, suggesting that a more rigorous validation for some antibodies is needed, perhaps on a tissue‐specific basis. Lastly, the WCHS is a single retrospective cohort study and our findings require replication in additional populations of Black and White women with breast cancer.

## 5 Conclusions

In summary, the TME in breast cancer contains immune infiltrates that have important and diverse roles in oncogenesis, disease progression, and immunotherapy response. We validated the DSP platform as an innovative method for highly multiplexed immune profiling in a population of Black and White women with invasive breast cancer. DSP was used for discovery and conventional IHC and gene expression data were used for validation. We found the DSP technology to be efficient, reproducible, and yield results that were largely concordant with other platforms, but showed disagreement with study‐specific findings, possibly due to analyte‐specific technical factors. We observed significant inverse associations between ER and several immune markers. Lastly, B7‐H3 was significantly lower in Black women, an intriguing finding that should be further evaluated in independent and larger patient populations.

## Conflict of interest

The authors declare no conflict of interest.

## Author contributions

The study was designed, directed, and coordinated by ARO, CBA, and SY. ARO, CCH, EVB, CBA, and SY acquired the data. ARO, HS, and SY analyzed and interpreted the data. TK provided pathology assistance with image interpretation, samples, and TMA construction. ARO and SY wrote the manuscript and all authors reviewed and approved the work.

## Supporting information


**Fig. S1**. Heatmap of 33 DSP markers in 159 patient samples before correction for batch effect. Batch 1 consisted of freshly cut TMA sections whereas TMAs from Batch 2 were sectioned previously and stored in a desiccator. The heatmap showed significant batch effect.

**Fig. S2**. Principal component analysis (PCA) plots of the DSP data demonstrating a batch effect for two staining batches: (A) before correction, (B) after correction. Batch 1 consisted of freshly cut TMA sections whereas TMAs from Batch 2 were sectioned previously and stored in a desiccator. The comparison of (A) and (B) indicated that the correction was successful.

**Fig. S3**. Kaplan–Meier plots of OS (defined as the time from diagnosis to the date of the last contact or of death from any causes) by dichotomized (at the median) DSP markers in the tumor compartment. The *P*‐values from the log‐rank test were reported. A subset of the study population with available follow‐up data was used (*N* = 136).

**Fig. S4**. Kaplan–Meier plots of OS (defined as the time from diagnosis to the date of the last contact or of death from any causes) by dichotomized (at the median) DSP markers in the stromal compartment. The *P*‐values from the log‐rank test were reported. A subset of the study population with available follow‐up data was used (*N* = 136).

**Fig. S5**. Kaplan–Meier plots of breast cancer specific survival (defined as the time from diagnosis to the date of the last contact or of death from breast cancer) by dichotomized (at the median) DSP markers in the tumor compartment. The *P*‐values from the log‐rank test were reported. A subset of the study population with available follow‐up data was used (*N* = 136).

**Fig. S6**. Kaplan–Meier plots of breast cancer specific survival (defined as the time from diagnosis to the date of the last contact or of death from breast cancer) by dichotomized (at the median) DSP markers in the stromal compartment. The *P*‐values from the log‐rank test were reported. A subset of the study population with available follow‐up data was used (*N* = 136).

**Table S1**. Complete list of protein targets used in the NanoString DSP assay. Nineteen analytes with more than 50% of values having an SNR < 3 in either the tumor or the stromal compartment were deleted from our analysis and marked with an asterisk. Positive control (PC) and negative control (NC) markers are also noted.
**Table S2**. Complete dataset for all participants and markers analyzed in this study.
**Table S3**. Median analyte scores and interquartile range for HER2‐positive vs HER2‐negative cases. HER2 status was determined from the patients' pathology reports. The *P*‐values from two sample *t*‐test were corrected for FDR and reported as *q*‐values using Benjamini‐Hochberg method to account for multiple testing.

**Table S4**. Median analyte scores (IQR) for Black and white women. ANCOVA was conducted on the associations between race and marker values, adjusted for breast cancer subtype, grade, and BMI. The main effect of race was tested using sum of squares by *F*‐test. Model assumptions were checked using QQ plot and residual plots and *P*‐values were corrected for FDR and reported as *q*‐values using Benjamini‐Hochberg method to account for multiple testing.

**Table S5**. Median analyte scores (IQR) for Black and white women. ANOVA was conducted on the associations between race and marker values, without adjustment for other factors. The main effect of race was tested using sum of squares by *F*‐test. Model assumptions were checked using QQ plot and residual plots and *P*‐values were corrected for FDR and reported as *q*‐values using Benjamini‐Hochberg method to account for multiple testing.

## Data Availability

The dataset analyzed for this study is included as Table S2.
