# Supplementary figures and images for: Multiplexed digital spatial profiling of invasive breast tumors from Black and White women
Source: Mol Oncol. 2021 Jun 10;16(1):54–68. doi: 10.1002/1878-0261.13017 (PMC8732343; doi:10.1002/1878-0261.13017)

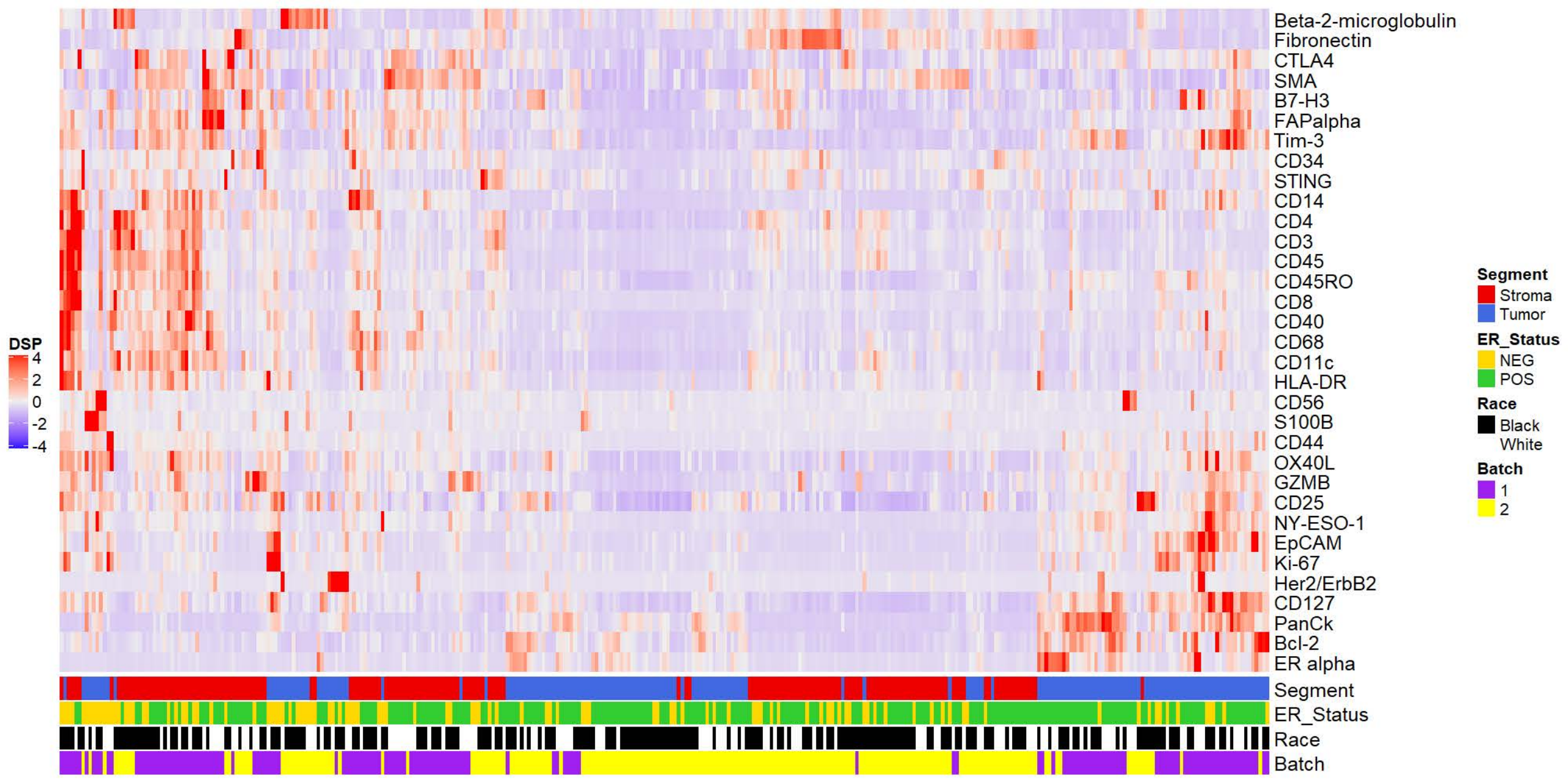

Supplement: Supplementary file 1 — Fig. S1. Heatmap of 33 DSP markers in 159 patient samples before correction for batch effect. Batch 1 consisted of freshly cut TMA sections whereas TMAs from Batch 2 were sectioned previously and stored in a desiccator. The heatmap showed significant batch effect. [file MOL2-16-54-s003.pdf]

**A**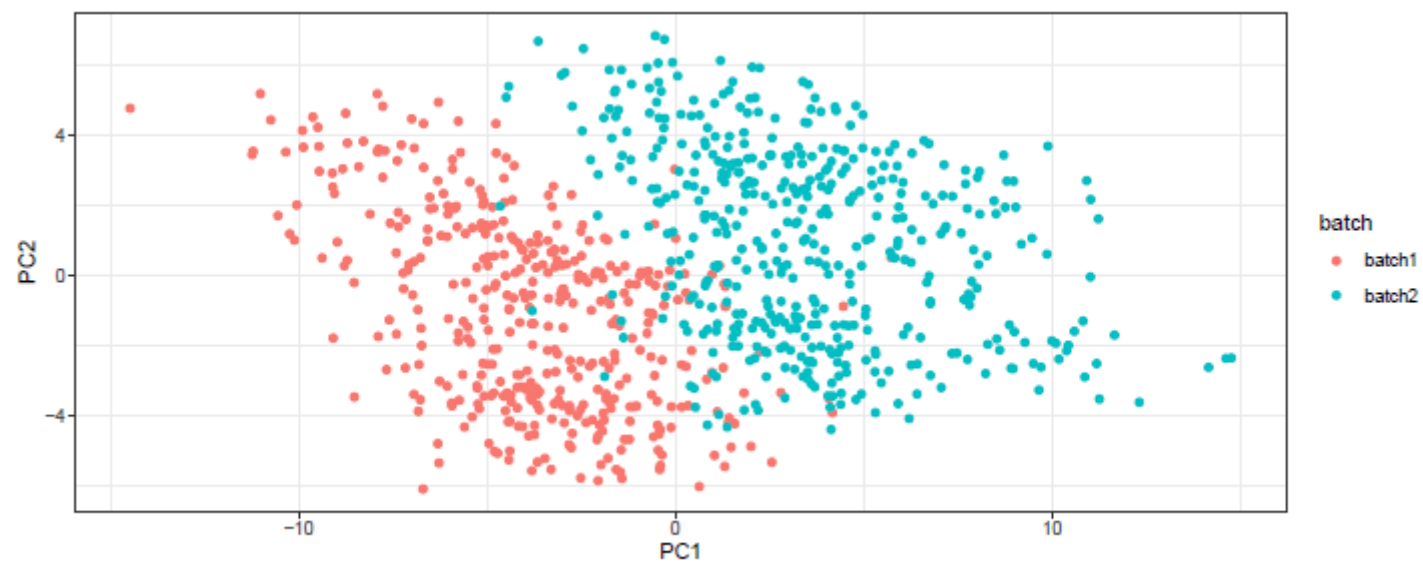**B**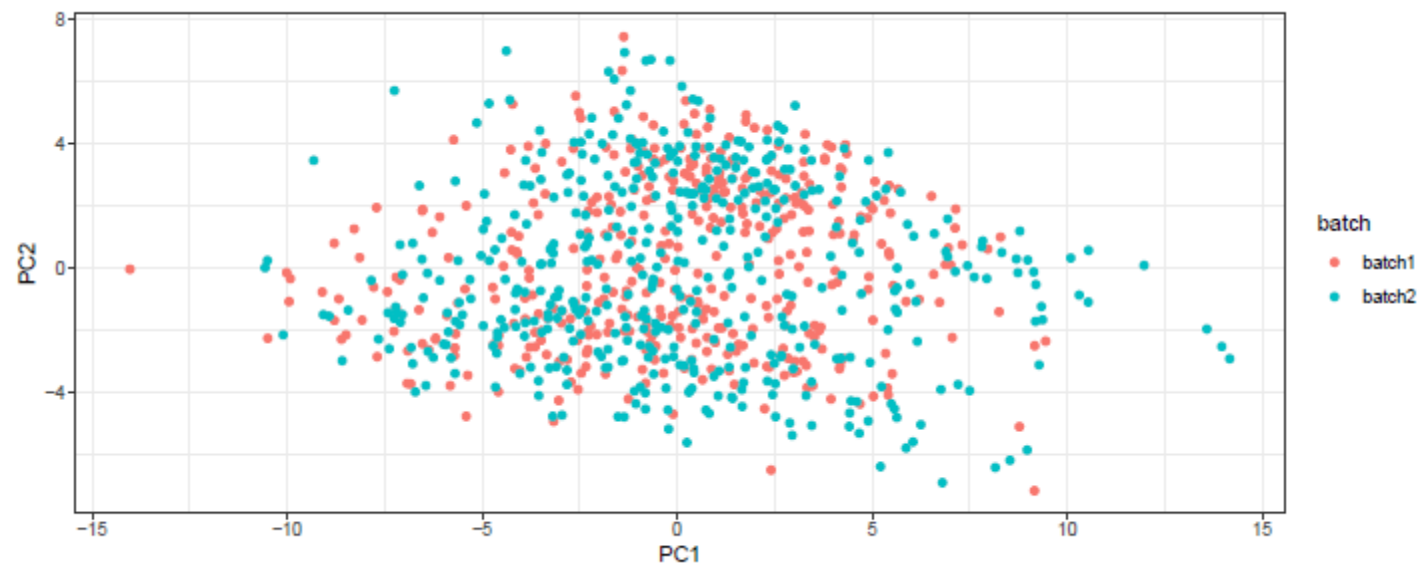

Supplement: Supplementary file 2 — Fig. S2. Principal component analysis (PCA) plots of the DSP data demonstrating a batch effect for two staining batches: (A) before correction, (B) after correction. Batch 1 consisted of freshly cut TMA sections whereas TMAs from Batch 2 were sectioned previously and stored in a desiccator. The comparison of (A) and (B) indicated that the correction was successful. [file MOL2-16-54-s009.pdf]

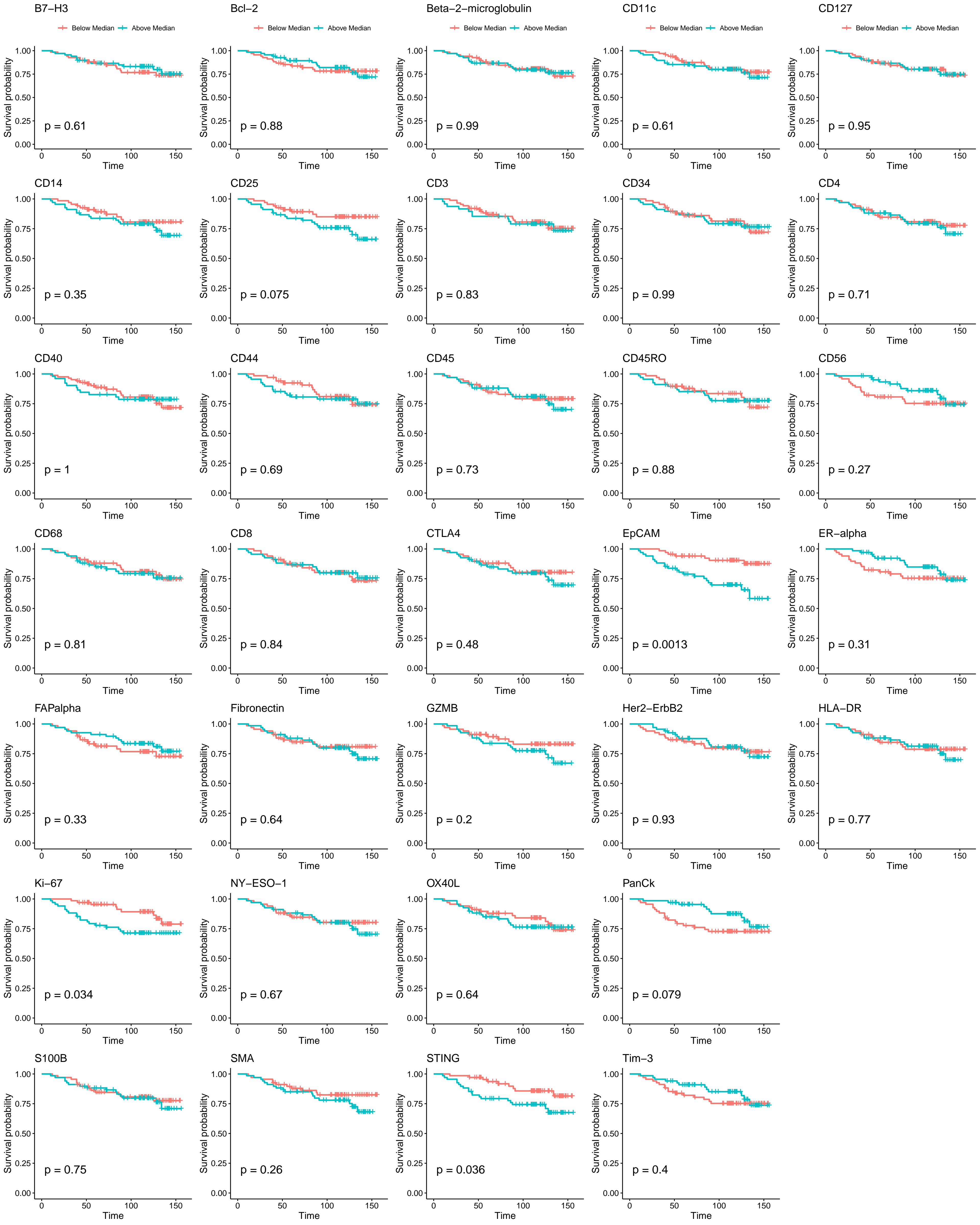

Supplement: Supplementary file 3 — Fig. S3. Kaplan–Meier plots of OS (defined as the time from diagnosis to the date of the last contact or of death from any causes) by dichotomized (at the median) DSP markers in the tumor compartment. The P‐values from the log‐rank test were reported. A subset of the study population with available follow‐up data was used (N = 136). [file MOL2-16-54-s001.pdf]

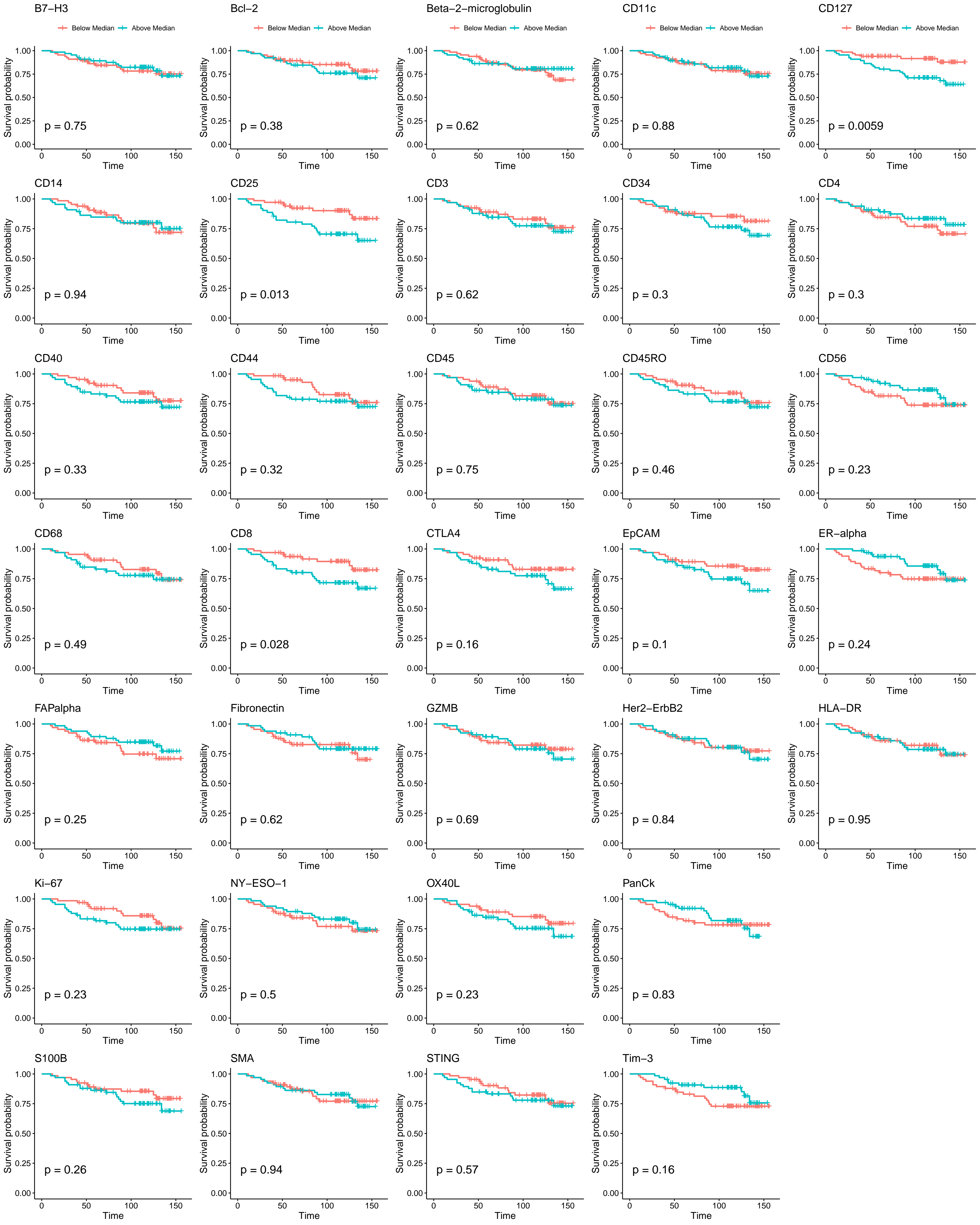

Supplement: Supplementary file 4 — Fig. S4. Kaplan–Meier plots of OS (defined as the time from diagnosis to the date of the last contact or of death from any causes) by dichotomized (at the median) DSP markers in the stromal compartment. The P‐values from the log‐rank test were reported. A subset of the study population with available follow‐up data was used (N = 136). [file MOL2-16-54-s005.pdf]

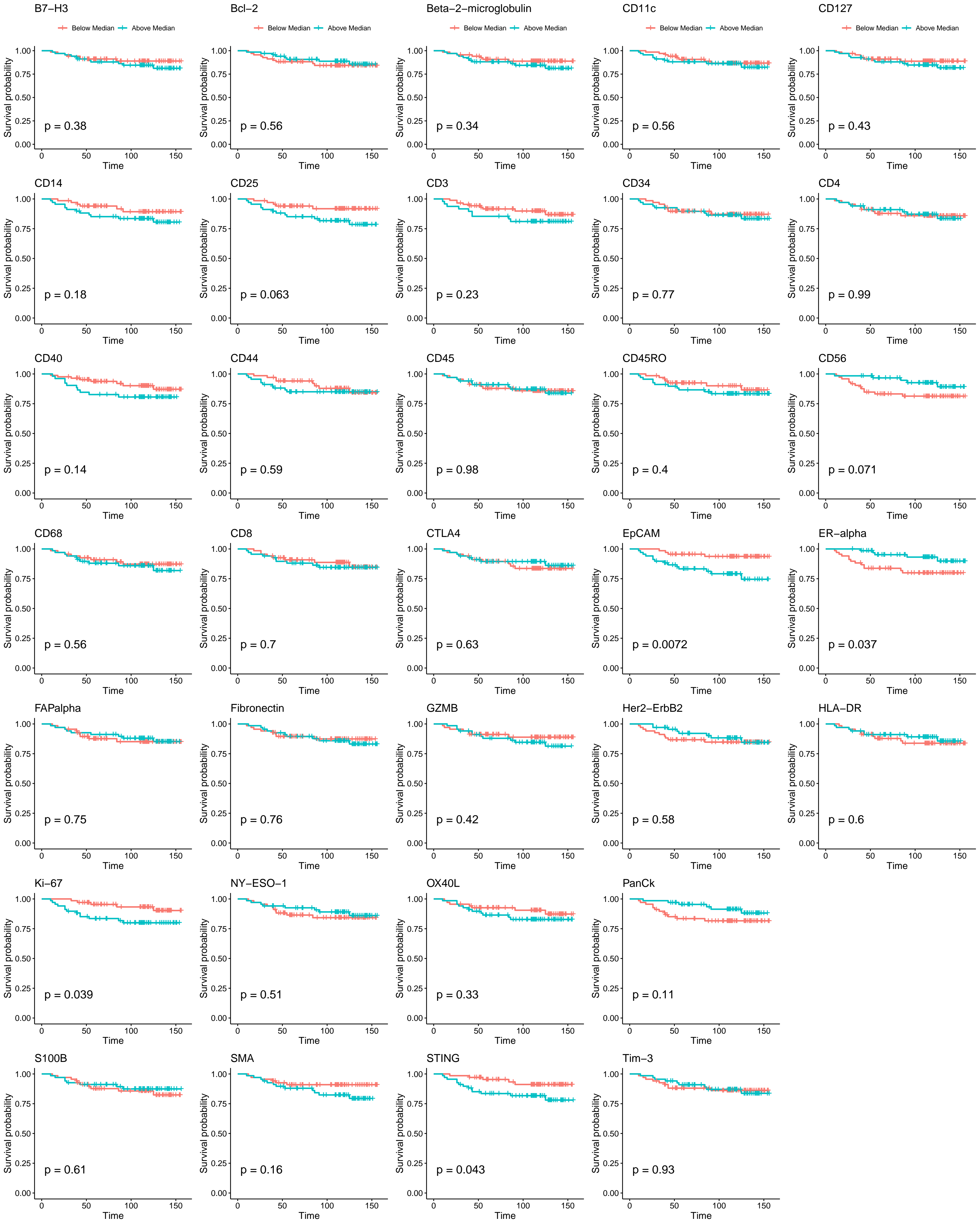

Supplement: Supplementary file 5 — Fig. S5. Kaplan–Meier plots of breast cancer specific survival (defined as the time from diagnosis to the date of the last contact or of death from breast cancer) by dichotomized (at the median) DSP markers in the tumor compartment. The P‐values from the log‐rank test were reported. A subset of the study population with available follow‐up data was used (N = 136). [file MOL2-16-54-s008.pdf]

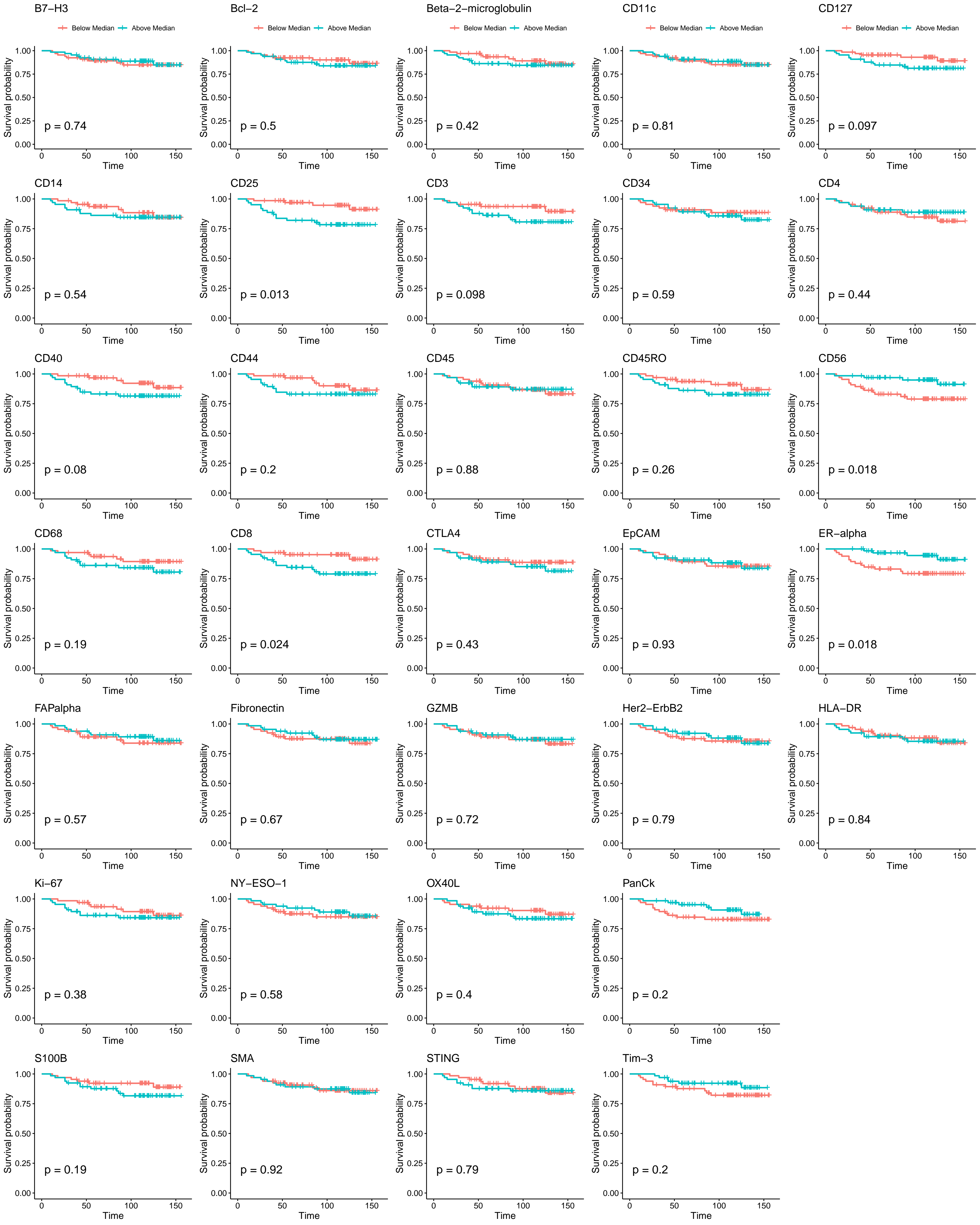

Supplement: Supplementary file 6 — Fig. S6. Kaplan–Meier plots of breast cancer specific survival (defined as the time from diagnosis to the date of the last contact or of death from breast cancer) by dichotomized (at the median) DSP markers in the stromal compartment. The P‐values from the log‐rank test were reported. A subset of the study population with available follow‐up data was used (N = 136). [file MOL2-16-54-s006.pdf]
