# Supplementary material for: Multiplexed digital spatial profiling of invasive breast tumors from Black and White women
Source: Mol Oncol. 2021 Jun 10;16(1):54–68. doi: 10.1002/1878-0261.13017 (PMC8732343; doi:10.1002/1878-0261.13017)
Supplement: Supplementary file 7 — Table S1. Complete list of protein targets used in the NanoString DSP assay. Nineteen analytes with more than 50% of values having an SNR < 3 in either the tumor or the stromal compartment were deleted from our analysis and marked with an asterisk. Positive control (PC) and negative control (NC) markers are also noted. Table S2. Complete dataset for all participants and markers analyzed in this study. Table S3. Median analyte scores and interquartile range for HER2‐positive vs HER2‐negative cases. HER2 status was determined from the patients' pathology reports. The P‐values from two sample t‐test were corrected for FDR and reported as q‐values using Benjamini‐Hochberg method to account for multiple testing. [file MOL2-16-54-s007.docx]

Supplemental Table 1. Complete list of protein targets used in the NanoString DSP assay. Nineteen analytes with more than 50% of values having an SNR <3 in either the tumor or the stromal compartment were deleted from our analysis and marked with an asterisk. Positive control (PC) and negative control (NC) markers are also noted.

| Protein Name | Full Target name |
| --- | --- |
| 4-1BB CD137^*^ | tumor necrosis factor receptor superfamily, member 9 |
| ARG1^*^ | arginase 1 |
| B7-H3 | CD276 molecule |
| Bcl-2 | B-cell CLL/lymphoma 2 |
| β2-microglobulin | beta-2-microglobulin |
| CD11c | integrin, alpha X (complement component 3 receptor 4 subunit) |
| CD127 | interleukin 7 receptor |
| CD14 | CD14 molecule |
| CD163^*^ | CD163 molecule |
| CD20^*^ | membrane-spanning 4-domains, subfamily A, member 1 |
| CD25 | interleukin 2 receptor, alpha |
| CD27^*^ | CD27 molecule |
| CD3 | CD3g molecule, gamma (CD3-TCR complex), CD3e molecule, epsilon (CD3-TCR complex), CD3d molecule, delta (CD3-TCR complex) |
| CD34 | CD34 molecule |
| CD4 | CD4 molecule |
| CD40 | CD40 molecule, TNF receptor superfamily member 5 |
| CD44 | CD44 molecule (Indian blood group) |
| CD45 | protein tyrosine phosphatase, receptor type, C |
| CD45RO | protein tyrosine phosphatase, receptor type, C |
| CD56 | neural cell adhesion molecule 1 |
| CD66b^*^ | carcinoembryonic antigen-related cell adhesion molecule 8 |
| CD68 | CD68 molecule |
| CD8 | CD8a molecule |
| CD80^*^ | CD80 molecule |
| CTLA4 | cytotoxic T-lymphocyte associated protein 4 |
| EpCAM | epithelial cell adhesion molecule |
| ERα | estrogen receptor 1 |
| FAPα | fibroblast activation protein alpha |
| Fibronectin | fibronectin 1 |
| FOXP3^*^ | forkhead box P3 |
| GAPDH ^(PC)^ | glyceraldehyde-3-phosphate dehydrogenase |
| GITR^*^ | TNF receptor superfamily member 18 |
| GZMB | granzyme B |
| Her2 | v-erb-b2 avian erythroblastic leukemia viral oncogene homolog 2 |
| Histone H3 ^(PC)^ | histone 1, H3a |
| HLA-DR | CD74 molecule, major histocompatibility complex, class II invariant chain |
| ICOS^*^ | inducible T-cell co-stimulator |
| IDO1^*^ | indoleamine 2,3-dioxygenase 1 |
| Ki-67 | marker of proliferation Ki-67 |
| LAG3^*^ | lymphocyte-activation gene 3 |
| MART1^*^ | melan-A |
| Ms IgG1 ^(NC)^ | immunoglobulin G control |
| Ms IgG2a ^(NC)^ | immunoglobulin G control |
| NY-ESO-1 | cancer/testis antigen 1B |
| OX40L | tumor necrosis factor superfamily member 4 |
| PanCk | keratin 1, keratin 2, keratin 3, keratin 5, keratin 6A, keratin 6B, keratin 8, keratin 10, keratin 14, keratin 16, keratin 19 |
| PD-1^*^ | programmed cell death 1 |
| PD-L1^*^ | CD274 molecule |
| PD-L2^*^ | programmed cell death 1 ligand 2 |
| PR^*^ | progesterone receptor |
| PTEN^*^ | phosphatase and tensin homolog |
| Rb IgG ^(NC)^ | immunoglobulin G control |
| S100B | S100 calcium binding protein B |
| S6 ^(PC)^ | ribosomal protein S6 |
| SMA | actin, alpha 2, smooth muscle, aorta |
| STING | transmembrane protein 173 |
| Tim-3 | hepatitis A virus cellular receptor 2 |
| VISTA^*^ | V-set immunoregulatory receptor |

**Supplemental Table 3.** Median analyte scores and interquartile range for HER2-positive vs HER2-negative cases. HER2 status was determined from the patients’ pathology reports. The *p*-values from two sample *t*-test were corrected for false discovery rate (FDR) and reported as *q*-values using Benjamini-Hochberg method to account for multiple testing.

| **Marker** | **Tissue Compartment** | **HER2-positive** | **HER2-negative** | **q-value** |
| --- | --- | --- | --- | --- |
| B7-H3 | Tumor | 56.25 (71.81) | 33.14 (41.24) | 0.6618 |
| Bcl-2 | Tumor | 13.56 (25.1) | 23.45 (32.22) | 0.4458 |
| β2-microglobulin | Tumor | 7.18 (8.28) | 5.97 (7.2) | 0.9747 |
| CD11c | Tumor | 9.65 (3.56) | 10 (6.69) | 0.9747 |
| CD127 | Tumor | 80.11 (61.64) | 67.67 (48.31) | 0.9747 |
| CD14 | Tumor | 13.07 (16.26) | 14.99 (16.41) | 0.9747 |
| CD25 | Tumor | 3.76 (2.72) | 3.8 (3.69) | 0.9747 |
| CD3 | Tumor | 2.12 (0) | 2.12 (1.8) | 0.9747 |
| CD34 | Tumor | 7.45 (10.31) | 10.69 (8.23) | 0.9269 |
| CD4 | Tumor | 4.67 (3.14) | 6.17 (4.78) | 0.9747 |
| CD40 | Tumor | 2.12 (1.37) | 2.12 (1.82) | 0.9747 |
| CD44 | Tumor | 131.4 (224.5) | 134.36 (272.43) | 0.9269 |
| CD45 | Tumor | 12.74 (6.93) | 14.7 (17.17) | 0.9747 |
| CD45RO | Tumor | 4.83 (6.12) | 4.96 (3.64) | 0.9747 |
| CD56 | Tumor | 2.12 (1.55) | 2.12 (1.6) | 0.9747 |
| CD68 | Tumor | 16.35 (10.82) | 18.28 (13.12) | 0.9747 |
| CD8 | Tumor | 5.82 (3.84) | 7.25 (4.02) | 0.9747 |
| CTLA4 | Tumor | 5.74 (1.86) | 6.02 (4.69) | 0.9747 |
| EpCAM | Tumor | 85.82 (189.27) | 94.09 (141.92) | 0.9747 |
| ERα | Tumor | 17.05 (68.01) | 35.05 (75.79) | 0.9747 |
| FAPα | Tumor | 3.32 (3.92) | 3.7 (3.66) | 0.9747 |
| Fibronectin | Tumor | 31.27 (48.6) | 35.11 (45.04) | 0.9747 |
| GZMB | Tumor | 13.43 (6.14) | 15.15 (10.26) | 0.9747 |
| ***Her2*** | ***Tumor*** | ***273.18 (651.62)*** | ***9.44 (14.09)*** | ***0.0051*** |
| HLA-DR | Tumor | 13.27 (9.92) | 15.87 (14.89) | 0.6618 |
| Ki-67 | Tumor | 14.78 (15.35) | 13.88 (19.04) | 0.9747 |
| NY-ESO-1 | Tumor | 7.99 (5.43) | 8.31 (6.11) | 0.9269 |
| OX40L | Tumor | 4.06 (3.74) | 3.81 (4.57) | 0.9747 |
| PanCk | Tumor | 435.09 (310.98) | 441.63 (447.83) | 0.9747 |
| S100B | Tumor | 3.91 (6.37) | 9.82 (18.84) | 0.9747 |
| SMA | Tumor | 354.14 (284.22) | 397.27 (398.02) | 0.9747 |
| STING | Tumor | 8.13 (4.71) | 12.07 (8.1) | 0.4458 |
| Tim-3 | Tumor | 27.79 (15.55) | 28.27 (26.34) | 0.9747 |
| B7-H3 | Stroma | 57.63 (66.68) | 37.09 (48.07) | 0.6996 |
| Bcl-2 | Stroma | 6.97 (5.15) | 9.32 (6.49) | 0.3900 |
| β2-microglobulin | Stroma | 11.23 (6.97) | 8.93 (8.77) | 0.6828 |
| CD11c | Stroma | 27.67 (28.69) | 22.99 (21.88) | 0.6996 |
| CD127 | Stroma | 29.2 (21.03) | 30.5 (19.99) | 0.9888 |
| CD14 | Stroma | 40.5 (35.47) | 24.59 (41.11) | 0.6828 |
| CD25 | Stroma | 2.12 (2.76) | 2.57 (2.52) | 0.9830 |
| CD3 | Stroma | 7.66 (7.68) | 6.26 (8.83) | 0.8760 |
| CD34 | Stroma | 18.13 (15.13) | 23.38 (26.03) | 0.4652 |
| CD4 | Stroma | 15.67 (10.05) | 12.33 (13.63) | 0.6996 |
| CD40 | Stroma | 6.3 (6.11) | 4.67 (6.26) | 0.6996 |
| CD44 | Stroma | 204.47 (220.35) | 153.11 (203.06) | 0.9093 |
| CD45 | Stroma | 40.93 (48.32) | 38.48 (52.96) | 0.9093 |
| CD45RO | Stroma | 5.86 (6.54) | 5.84 (4.96) | 0.6996 |
| CD56 | Stroma | 3.01 (1.94) | 3.2 (1.51) | 0.8523 |
| CD68 | Stroma | 50.82 (32.77) | 37.12 (33.63) | 0.7800 |
| CD8 | Stroma | 12.33 (7.95) | 12.66 (13.37) | 0.8679 |
| CTLA4 | Stroma | 7.65 (6.29) | 5.98 (7.74) | 0.4872 |
| EpCAM | Stroma | 16.98 (18.05) | 17.67 (23.85) | 0.9784 |
| ERα | Stroma | 3.36 (3.86) | 3.81 (6.7) | 0.6828 |
| FAPα | Stroma | 7.93 (7.55) | 6.63 (6.45) | 0.7186 |
| Fibronectin | Stroma | 197.2 (128.28) | 173.62 (123.36) | 0.9093 |
| GZMB | Stroma | 11.89 (11.26) | 15.43 (15.08) | 0.4652 |
| ***Her2*** | ***Stroma*** | ***25.86 (79.43)*** | ***2.12 (2.44)*** | ***0.0207*** |
| HLA-DR | Stroma | 34.17 (33.98) | 38.47 (40.18) | 0.9093 |
| Ki-67 | Stroma | 5.09 (6.35) | 5.73 (4.61) | 0.9784 |
| NY-ESO-1 | Stroma | 3.55 (2.33) | 4.01 (2.74) | 0.3900 |
| OX40L | Stroma | 3.94 (2.48) | 3.89 (3.84) | 0.6996 |
| PanCk | Stroma | 14.06 (26.76) | 23.38 (32.25) | 0.6996 |
| S100B | Stroma | 5.63 (11.45) | 11.88 (26.04) | 0.9830 |
| SMA | Stroma | 1305.8 (1122.73) | 1248.24 (1018.91) | 0.9093 |
| STING | Stroma | 14.92 (13.69) | 15.3 (12.6) | 0.9093 |
| Tim-3 | Stroma | 29.24 (24.23) | 29.36 (17.35) | 0.9784 |
